# Antiproliferative Evaluation of Dextran Polymer-Based Pomegranate Ethanolic Extract

**DOI:** 10.3390/ijms262110618

**Published:** 2025-10-31

**Authors:** Umile Gianfranco Spizzirri, Marisa Francesca Motta, Sonia Ferraro, Silvia Strigaro, Cinzia Benincasa, Rosa Nicoletti, Francesco Astuto, Ubaldo Comite, Rocco Malivindi, Francesca Aiello

**Affiliations:** 1 Ionian Department of Law, Economics and Environment, University of Bari Aldo Moro, 74123 Taranto, Italy; umile.spizzirri@uniba.it (U.G.S.); astutof22@gmail.com (F.A.); 2 Department of Pharmacy, Health and Nutritional Sciences, University of Calabria, Edificio Polifunzionale, 87036 Rende, Italy; marisafrancesca.motta@unical.it (M.F.M.); ferraro24sonia@gmail.com (S.F.); silvia.strigaro@libero.it (S.S.); francesca.aiello@unical.it (F.A.); 3 Council for Agricultural Research and Economics (CREA), Research Centre for Olive, Fruit and Citrus Crops, Via Settimio Severo 83, 87036 Rende, Italy; cinzia.benincasa@crea.gov.it (C.B.); rosa.nicoletti@crea.gov.it (R.N.); 4 Department of Business Sciences, University Giustino Fortunato, 82100 Benevento, Italy; u.comite@unifortunato.eu; 5 Clinical Laboratory Unit, AO SS Annunziata, 87100 Cosenza, Italy

**Keywords:** pomegranate peel, bioactive compounds, antioxidant activity, breast cancer, dextran grafting

## Abstract

The pomegranate peel represents an important source of secondary metabolites such as hydrolysable ellagitannins, which are recognized for their antioxidant, anticancer and neuroprotective properties. In this work, the freeze-dried pomegranate peel was extracted by a combined mild maceration at room temperature and ultrasonication at 45 °C using ethanol and acetone as green solvents. The ethanol extract, with an extraction yield of 29%, and IC50 (mg/mL) 0.1067 and 0.0414 for DPPH and ABTS, respectively, was incorporated into a polymer based on dextran, using a grafting reaction, to improve its bioavailability and preserve the chemical integrity. In addition, the potential antitumor activity against breast cancer was evaluated based on the existing literature. In vitro studies have demonstrated the safety and biocompatibility of both free pomegranate peel extract (SSE2-L) and its dextran conjugate (SSPD), with no adverse effects on fibroblasts, erythrocytes, or immune cells. Both formulations inhibited the proliferation of breast cancer cell lines (MCF-7, MDA-MB-231) in a concentration- and time-dependent manner, with SSPD consistently showing superior efficacy. This enhanced activity was corroborated by reduced clonogenic growth, G1 cell-cycle arrest, and improved stability and bioactive retention conferred by polymer conjugation. Overall, these findings highlight dextran-conjugated pomegranate polyphenols as promising candidates for next-generation nutraceuticals and phytopharmaceuticals in cancer chemoprevention and adjunctive therapy, with potential applications extending to other biomedical fields and functional foods.

## 1. Introduction

Pomegranate (*Punica granatum* L.) is a perennial fruit-bearing shrub belonging to the family Lythraceae, cultivated extensively in arid and semi-arid regions worldwide. Traditionally valued for its nutritional, medicinal, and sensory qualities, it has attracted increasing scientific attention due to its wide spectrum of bioactivities, including antioxidant, anti-inflammatory, antimicrobial, cardioprotective, and anticancer effects [1,2]. Nevertheless, commercial pomegranate processing, particularly for juice production, generates substantial quantities of agro-industrial waste. The edible arils account for only 38–45% of the fresh fruit mass, while the peel constitutes 45–50% and the seeds 10–15% [3]. Consequently, global juice production is associated with the generation of approximately one million tons of peels and 200,000 tons of seeds annually, which are often discarded or utilized as low-value by-products despite their richness in bioactive compounds [3,4].

Pomegranate peel is an especially rich source of polyphenols, which account for about 92% of the total antioxidant activity of the fruit [5,6]. Among these, hydrolysable tannins, predominantly ellagitannins such as punicalagin, punicalin, and granatin A and B, are the most abundant and biologically relevant constituents [7]. These compounds display potent antioxidant capacity and have been associated with a range of beneficial effects, including antiproliferative, anti-angiogenic, and neuroprotective properties [8]. However, their pharmacokinetic profile poses significant challenges. Ellagitannins are poorly absorbed in their native form due to their high molecular weight and hydrophilicity [9]. During gastrointestinal transit, they undergo hydrolysis to ellagic acid, which is further metabolized by the intestinal microbiota into urolithins-dibenzopyran-6-one derivatives that are more lipophilic, exhibit greater bioavailability, and have longer plasma half-lives than their precursors [10,11]. These metabolites have been detected in plasma and peripheral tissues, including colon, prostate, and breast [12]. Nonetheless, urolithin production is highly variable between individuals due to differences in gut microbiota composition, potentially influencing the consistency and magnitude of the biological response [9].

A growing body of in vitro and in vivo evidence supports the anticancer potential of pomegranate peel polyphenols. Punicalagin, ellagic acid, and related metabolites have demonstrated the ability to inhibit cancer cell proliferation, induce apoptosis, arrest the cell cycle at G0/G1, suppress epithelial–mesenchymal transition, and inhibit angiogenesis in diverse cancer models [8,13]. Their activity has been reported against multiple tumor types, including breast, colon, bladder, prostate, thyroid, liver, leukemia, and osteosarcoma [7,14,15,16]. Notably, ellagitannins have been shown to enhance the cytotoxicity of chemotherapeutic agents such as 5-fluorouracil in murine colorectal cancer models, while mitigating chemotherapy-induced toxicity [7]. Despite these promising findings, the clinical translation of pomegranate polyphenols is hindered by their chemical instability, low and variable bioavailability, and rapid metabolic clearance [17,18,19].

Recent advances in drug delivery systems have explored the use of nanocarriers and functionalized biopolymers to overcome these limitations by protecting bioactive molecules from premature degradation, enhancing gastrointestinal absorption, and enabling sustained or targeted release [20,21]. Among natural polymers, dextran has emerged as a particularly promising carrier due to its biocompatibility, biodegradability, ease of chemical modification, and widespread use in pharmaceutical and biomedical formulations [22]. Covalent grafting of bioactives onto dextran can improve aqueous solubility, reduce susceptibility to hydrolytic and enzymatic degradation, and modulate pharmacokinetic behavior for more effective delivery [23].

In this study, freeze-dried pomegranate peel was extracted using a combined maceration–ultrasonication method with green solvents, producing ethanolic and acetonic extracts. The ethanolic extract, which demonstrated superior yield and antioxidant activity, was selected for covalent grafting onto a dextran backbone, generating a functionalized biopolymer designed to protect ellagitannins, enhance their stability, and facilitate controlled release. The antiproliferative activity of this dextran-conjugated extract was evaluated in human breast cancer cell lines (MCF-7 and MDA-MB-231) to assess its potential as a sustainable, plant-derived therapeutic platform for anticancer applications.

## 2. Results

### 2.1. Extracts from Pomegranate Peels

The application of a mixed method, maceration of 18 h at room temperature and subsequent sonication for 40 min at 45 °C allowed to obtain extracts rich of bioactive substances, SSA1 (oven-dried peels using acetone as solvent), and SSE2 (oven-dried peels using ethanol as solvent), SSA1-L (freeze dried peels using acetone as solvent), and SSE2-L (freeze dried peels using ethanol as solvent). This finding is consistent with the results reported by Liu et al. (2022), who employed ultrasonic-assisted extraction to recover punicalagin from pomegranate peel [24]. In their study, ultrasound application enhanced mass transfer and facilitated the rupture of plant cell walls, leading to a higher extraction yield compared to conventional maceration. The optimized conditions (extraction temperature, solvent concentration, and ultrasonic power) enabled the isolation of punicalagin with a high purity level. This suggests that ultrasonic-assisted extraction not only improves the recovery efficiency of bioactive ellagitannins but also preserves their structural integrity and functional properties. The alignment of our results with these literature data reinforces the suitability of ultrasound as a green, efficient, and selective extraction technique for pomegranate peel polyphenols. In particular, the use of ethanol as the extraction solvent provided a significantly higher yield (29.4% for the lyophilized sample) compared to acetone (7.24% for the lyophilized sample), confirming the superior extraction efficiency of the alcoholic solvent with respect to the acetone. These results are in line with the findings of Liu et al. (2022) [24], who also reported ethanol as the most effective solvent for ultrasonic-assisted extraction of punicalagin from pomegranate peel, achieving a markedly greater recovery compared to other tested solvents. In both studies, TLC following a literature-reported procedure, further supporting ethanol’s ability to efficiently solubilize and preserve these high-molecular-weight phenolic compounds during extraction [25,26], confirmed the predominance of hydrolysable tannins in the extracts, in our case.

### 2.2. ESI-MS/MS Characterization of SSE2-L and SSPD Matrices

Qualitative ESI-MS/MS analysis was conducted both on the extract (SSE2-L) and on the functionalized polymer (SSPD). To verify the success of the molecular grafting reaction, a control analysis was performed on the dextran polymer synthesized without extract (BDX). The full ion scans of BDX, SSE2-L and SSPD are shown in Supplementary Materials Figures S1–S3, respectively. The mass spectrum related to SSE2-L showed the characteristic peaks of the pomegranate secondary metabolic profile. More specifically, *m/z* ratios at 1084.0, 951.6 and 781.5 indicate the presence of ellagic acid derivatives, such as punicalgin, granatin B and punicalin, phenols belonging to the ellagitannins subgroup. This profile is consistent with the findings of Sentandreu et al. (2013), who analyzed pomegranate juice by HPLC-DAD-ESI-MS^n^ and reported similar ellagitannin-related ions, with total ellagitannin contents ranging from approximately 2010 to 6420 mg/L, confirming punicalagin as the predominant compound [27]. Likewise, Qu et al. (2012) quantitatively determined punicalagin α and β isomers, along with ellagic acid and gallic acid, in various pomegranate products, obtaining high recovery rates (92.4–95.5%) and further demonstrating the prevalence of punicalagin as the major phenolic constituent [28]. The strong agreement between our results and these studies highlights the reproducibility of the pomegranate phenolic fingerprint across different matrices and analytical platforms.

From the comparison of SSE2-L and SSPD mass spectra (see Supplementary Materials), it is evident that the molecular grafting reaction between the dextran and the extract itself took place successfully. The profiles of the two spectra, in fact, are practically superimposable for the peaks related to the protonated molecular ions up to *m*/*z* 500. Therefore, to obtain structural information on the common and most represented peaks present in SSE2-L and SSPD mass spectra, product ion scan measurements were performed. The deprotonated molecular ions and their main fragments are summarized in Table S1.

### 2.3. Evaluation of the Phenolic Profile and Antioxidant Activity of the Extracts

The colorimetric assays conducted enabled a comparison of the phenolic profile and antioxidant activity of each extract (Table 1).

The data obtained enabled the identification of the most effective drying method and solvent to produce an extract with enhanced antioxidant activity, while also minimizing the environmental impact of its production. According to the data in Table 1, the TPC of the extracts obtained from oven-dried matrices, SSA1 (19.53 ± 0.84 mg GAE/g) and SSE2 (19.43 ± 0.89 mg GAE/g), are comparable and do not show significant differences. However, it is evident that the TPC values of SSA1 and SSE2 are significantly lower than those of SSA1-L (466.12 ± 16.24 mg GAE/g) and SSE2-L (318.08 ± 12.85 mg GAE/g). This difference can likely be attributed to the extraction temperature, suggesting that heat negatively affects the preservation of the phenolic molecules in the extracts [29]. Literature data confirmed the same order of magnitude of the TPC value (105–470 mg GAE/g) for peel pomegranate extracts produced by natural deep eutectic solvents combined with ultrasound-assisted extraction and pressurized liquid extraction [30]. Similarly, the TPC of sonicated peel extracts from different cultivars of pomegranate were analyzed and the recorded values ranged from 52.91 to 134.16 mg GAE/g [31]. Alternatively, pomegranate peels were extracted through maceration at room temperature, yielding TPC values ranging from 276 to 416 mg GAE/g [32]. This study also highlights that, among the components of pomegranate, peel stands out as the primary source of polyphenols compared to the seed and juice. The same authors also quantified the total flavonoid content in pomegranate peels, reporting values equal to 36–54 mg rutin/g [33]. However, a direct comparison with our data was not feasible due to the use of different units. In our experiment, the FC data recorded for SSE2 was 31.32 ± 1.24 mg CTE/g, whereas the FC value for SSA1-L was more than an order of magnitude higher (365.23 ± 15.41 mg CTE/g).

Among all the samples, SSA1-L exhibited the lowest IC50 value (0.0093 mg mL^−1^) against the ABTS radical species. However, it is worth noting that the ethanolic extract SSE2-L, despite having a lower TPC and reduced inhibitory capacity against the ABTS radical (0.0191 mg mL^−1^, about two times higher), demonstrated the highest inhibitory capacity against the DPPH radical (0.0159 mg mL^−1^, −15%), performing more effectively in an organic environment. These findings align with the results of Elfalleh et al. (2012), who reported that the scavenging activity of pomegranate peels in both organic and aqueous environments is closely influenced by the extraction solvent [33]. Specifically, the use of an alcoholic solvent enhances the extract’s performance in an organic medium (0.00388 vs. 0.01148 mg mL^−1^) while reducing its scavenging activity in an aqueous medium (0.00380 vs. 0.00750 mg mL^−1^). These findings, combined with the fact that ethanol as the extraction solvent ensured a significantly higher extraction yield, led to the selection of SSE2-L for the subsequent synthesis of the DX polymeric conjugate.

### 2.4. Synthesis and Antioxidant Activity of Dextran Conjugate via Grafting Reaction

The synthesis of the grafted conjugate between the dextran polymer chains and the active molecules in the SSE2-L extract, referred to as SSPD, was carried out using a literature methodology with some changes [34]. The interaction between the biopolymer and the phytocompounds within natural extracts leads to synergistic effects, resulting in enhanced characteristics compared to the non-functionalized polymer or isolated biomolecules, increasing the overall effectiveness of the final material [35]. In synthesizing the polymer conjugate, it is crucial to prioritize techniques that preserve the integrity of natural compounds by avoiding invasive conditions, while also adopting environmentally friendly methods that minimize toxic solvents and energy consumption, ensuring a lower environmental impact [36].

The DX conjugate (SSPD) was synthesized via grafting reaction, using ascorbic acid and hydrogen peroxide as a redox initiator pair. The chemical process takes place in an aqueous environment, at low temperatures, and avoids the formation of any toxic intermediates [37]. The redox pair interaction involves the oxidation of ascorbic acid by hydrogen peroxide, generating hydroxyl and ascorbate radicals. These radicals initiate the polymerization reaction, primarily targeting polyphenol molecules at the ortho position relative to the hydroxyl group [38]. To assess the effectiveness of the grafting procedure, a blank polymer (BDX) was prepared under identical conditions, but without the addition of the extract.

Antioxidant characterization of SSPD displayed the success of the grafting reaction, returning TPC and FC values equal to 26.53 ± 1.12 mg GAE/g and 47.50 ± 1.89 mg CTE/g, respectively. These values appeared significantly lower than the data recorded for the extract due to the involvement of specific active molecules in the grafting process, because only selected active molecules in the phytochemical extract are involved in the grafting process. On the contrary, the same tests performed on BDX do not return any evident result. Additionally, the assessment of scavenging activity in both aqueous and organic environments highlighted the exceptional biological properties of the conjugate against radical species. The SSPD conjugate exhibited IC50 values of 0.1067 ± 0.0041 mg mL^−1^ against the DPPH radical and 0.0414 ± 0.0018 mg mL^−1^ against the ABTS radical, demonstrating significant scavenging activity in both environments.

### 2.5. Safety Assessment

#### 2.5.1. Cytotoxicity Evaluation: Neutral Red Uptake (NRU) Assay

**Figure 1:**
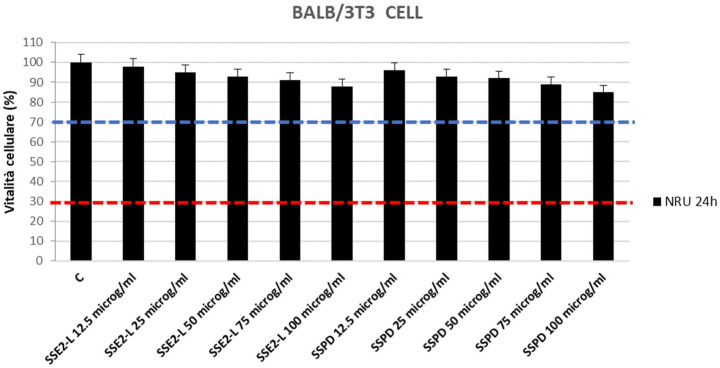
NRU assay outcomes. Percentage viability of BALB/3T3 Clone A31 fibroblasts determined using the neutral red uptake (NRU) cytotoxicity test after exposure to increasing concentrations of SSE2-L or SSPD. Data are expressed as mean ± SD from three replicate wells per group. The red dashed line indicates a strong cytotoxic effect (cell viability < 30%), whereas the blue dashed line marks the non-cytotoxic range (cell viability between 70% and 100%).

The toxicological safety of the synthesized compounds was investigated to assess the potential for cytotoxicity activity. The Neutral Red Uptake (NRU) Assay was performed according to guidelines ISO 10993-5:2009 [39] and ISO 10993-12:2021 [40]. The cellular layer was then examined for structural preservation, and biological reactivity, encompassing signs of degeneration or abnormal morphology, was scored according to ISO 10993-5:2009 [39] (Supplementary Materials Tables S2 and S3), which assigns a value from 0 to 4, with scores above 2 indicative of cytotoxicity. No sample produced values exceeding this threshold, suggesting that neither compound compromised cellular morphology under the tested conditions. Quantitative assessment was carried out via the Neutral Red Uptake (NRU) assay, which measures the ability of viable cells to sequester the supravital dye within functional lysosomes. Following exposure to graded concentrations of each compound, cultures were treated with Neutral Red, and dye retention was quantified by absorbance readings at 540 nm using a Synergy H1 microplate reader (Hybrid Reader, BioTek Instruments, Agilent, CA, USA). A decrease in cell viability greater than 30% relative to the untreated control (red line, Figure 1) was interpreted as evidence of cytotoxicity. The validity of the assay was supported by the performance of the positive control, which produced a reduction in viability of approximately 70% ± 10% (blue line, Figure 1).

Across all tested concentrations, both SSE2-L and SSPD maintained cell viability above the cytotoxicity threshold, in agreement with the morphological observations, thereby confirming their lack of detectable cytotoxic potential under the experimental conditions employed (Figure 1).

#### 2.5.2. In Vitro Analysis of Pro-Sensitizing Potential

The potential of the test substances to modulate immune-related co-stimulatory markers was assessed using the THP-1 monocyte cell line. 1 × 10^5^ cells were seeded and subsequently exposed for 24 h to the test samples at a concentration of 50 μg/mL, and the expression of CD54 and CD86, two key co-stimulatory molecules involved in the initiation of inflammatory and sensitization processes, was quantified by flow cytometry. Nickel sulfate (NiSO4), a well-characterized contact sensitizer, served as the positive control to confirm assay performance.

As expected, NiSO4 exposure resulted in a marked upregulation of both CD54 and CD86 compared to the negative control, with expression values exceeding the established cut-off thresholds (CD54 > 200; CD86 > 150), thereby validating the responsiveness of the assay system. Conversely, none of the tested products induced a statistically relevant modulation of either marker, with measured values remaining well below the defined cut-offs (Table 2 and Supplementary Materials Figure S4).

Cytotoxicity assessment indicated that the test samples did not adversely affect THP-1 cell viability under the conditions used, and no evidence of apoptosis was observed at the tested concentrations. These findings suggest that the evaluated materials are unlikely to trigger monocyte/macrophage-mediated immune activation through upregulation of co-stimulatory molecules, indicating an absence of detectable in vitro pro-sensitizing potential.

#### 2.5.3. Hemolytic Activity of SSE2-L and SSPD on Peripheral Blood

The potential hemolytic activity of SSE2-L and SSPD was assessed through a standard hemolysis assay using freshly collected peripheral blood from healthy adult volunteers. This assay is a well-established method to evaluate the cytotoxic potential of plant-derived products intended for medical or nutraceutical applications [41].

**Figure 2:**
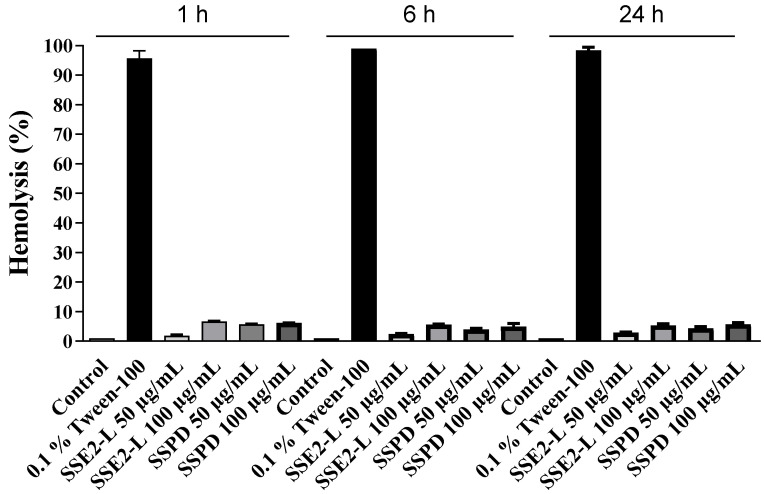
Hemolytic activity of biocompounds. Human red blood cells (RBCs) were incubated with phosphate-buffered saline (PBS; negative control), 0.1% Tween-100 (positive control), SSE2-L (50 or 100 μg/mL), or SSPD (50 or 100 μg/mL) for 1, 6, or 24 h. The histograms display the mean percentage of hemolysis relative to the positive control, obtained from three independent experiments, each conducted in triplicate. Statistical significance was determined in comparison with the 0.1% Tween-100 (positive control).

Peripheral blood samples were exposed to increasing concentrations of SSE2-L or SSPD, and the degree of hemolysis was determined spectrophotometrically by measuring hemoglobin release into the supernatant. As shown in Figure 2, none of the tested substances produced significant hemolysis compared with the negative control, with all values remaining well below the threshold indicative of a hemolytic effect.

These findings demonstrate that both SSE2-L and SSPD can be classified as non-hemolytic under the tested conditions, indicating a favorable safety profile with respect to erythrocyte membrane integrity.

### 2.6. Efficacy Assessment

#### Anti-Proliferative Response of Breast Cancer Cells to SSE2-L and SSPD Treatments

To determine whether the synthesized compounds exerted anti-proliferative activity against breast cancer cells, we employed three well-established human breast cancer cell models representing distinct molecular subtypes: MCF-7 cells (estrogen receptor-positive, metastatic ductal carcinoma) and MDA-MB-231 cells (triple-negative, metastatic adenocarcinoma). Proliferative capacity was evaluated using the anchorage-dependent MTT assay, which measures the metabolic conversion of 3-(4,5-dimethylthiazol-2-yl)-2,5-diphenyltetrazolium bromide into insoluble formazan crystals by mitochondrial dehydrogenases of viable cells.

2.5 × 10^4^ cells were seeded in a 48-well plate. After 24 h of incubation, cells were treated with the tested samples at doses of 12.5, 25, 50, 75, and 100 μg/mL [42]. After 24 h, a solution of MTT 0.2 mg/mL was added to each well and incubated for 3 h. After this period MTT solution was aspirated and formazan salts were solubilized by adding 200 μL of Dimethyl Sulfoxide (DMSO). Absorbance obtained was read at 570 nm using a Synergy H1 microplate reader (Hybrid Reader, BioTek Instruments, Agilent, CA, USA). The exposure to SSE2-L or SSPD produced a clear, concentration- and time-dependent reduction in MTT conversion across all tested cell lines, indicative of impaired metabolic activity and decreased cell viability. Notably, the inhibitory effect was consistently more pronounced in cultures treated with the pomegranate extract-associated polymeric compound compared to cells exposed solely to pomegranate extracts, suggesting a potentiating effect conferred by the polymeric component. This inhibitory trend was evident as early as the initial measurement time point and became progressively more substantial with increasing exposure duration and compound concentration.

**Figure 3:**
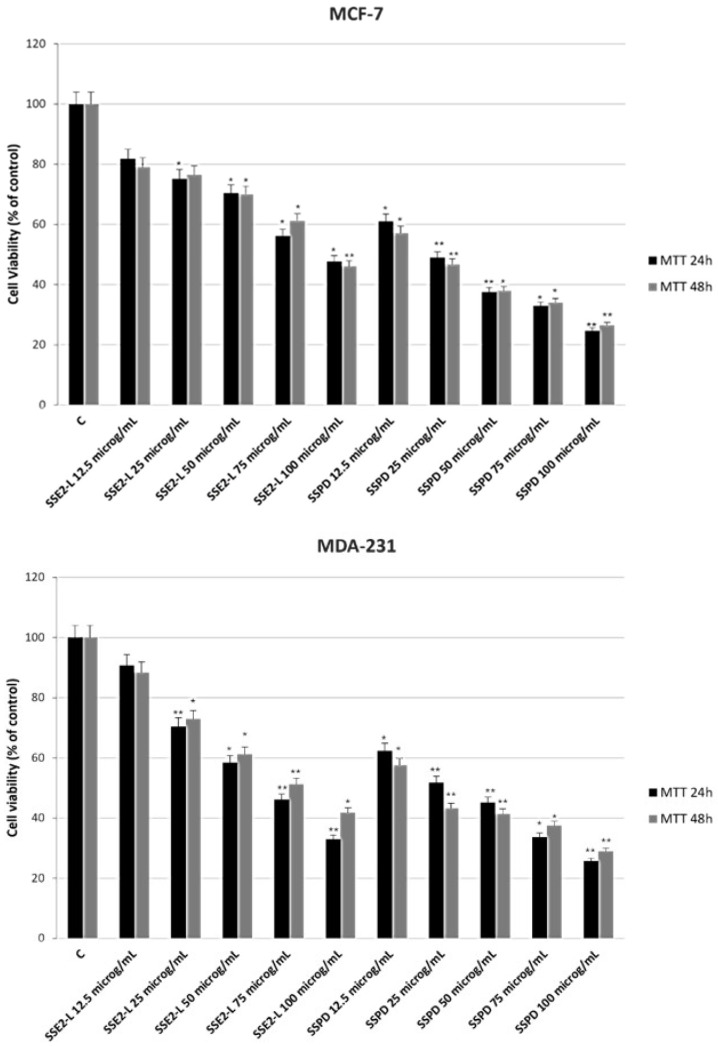
Evaluation of Cell Proliferation by MTT Assay in MCF-7 and MDA-MB-231 Breast Cancer Cells. MCF-7 and MDA-MB-231 breast cancer cells were exposed to escalating concentrations of the tested compounds for 24 h and 48 h, after which cell proliferation was quantified using the MTT colorimetric assay. Data are expressed as the percentage of mean absorbance relative to untreated controls and are presented as the mean ± SE from three independent experiments. Statistical analysis was performed using an unpaired two-tailed Student’s *t*-test. Significant differences from the control are denoted as * *p* < 0.05 and ** *p* < 0.005.

These results provide strong evidence that both SSE2-L and SSPD possess the capacity to significantly suppress proliferation in molecularly distinct breast cancer cell types. The observed anti-proliferative activity may be linked to interference with key cellular processes that sustain cancer cell growth and survival, although further mechanistic studies will be required to elucidate the underlying pathways. The quantitative data supporting these observations are presented in Figure 3, which illustrates the dose–response relationships and highlights the comparative potency of the tested formulations.

The inhibitory effects of the investigated compounds on the growth of MCF-7 and MDA-MB-231 breast cancer cells were further assessed using the anchorage-independent Soft Agar colony formation assay at a concentration of 50 μg/mL. At this dose, both cell lines treated with SSPD exhibited a reduction in proliferative capacity greater than that observed in cells exposed to SSE2-L, indicating a comparatively stronger potency of SSPD under these conditions.

**Figure 4:**
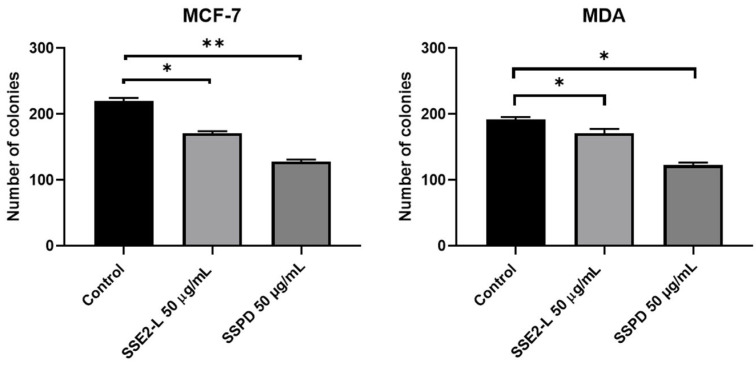
Effects of SSE2-L and SSPD on colony formation by MCF-7 and MDA-MB-231 cells. Colony formation assays were performed with MCF-7 and MDA-MB-231 cells (5 × 10^3^ cells per well) plated under basal conditions or in the presence of SSE2-L or SSPD. The relative colony formation rate was assessed after 14 days of culture. (* *p* < 0.05; ** *p* < 0.01 vs. basal).

Consistently, exposure to SSPD produced a more pronounced suppression of colony formation at the tested concentration over the 14-day treatment period in all cell lines examined (Figure 4). This outcome demonstrates that both formulations are able to impair long-term clonal expansion in a three-dimensional, anchorage-independent environment, a hallmark of tumorigenic potential; however, the superior inhibitory activity of SSPD highlights its enhanced efficacy in suppressing breast cancer cell clonogenicity compared with SSE2-L.

Taken together, these findings provide robust evidence that while both SSE2-L and SSPD interfere with anchorage-independent growth, SSPD displays a significantly higher capacity to suppress proliferation, suggesting that it may represent the more promising formulation for further development.

**Figure 5:**
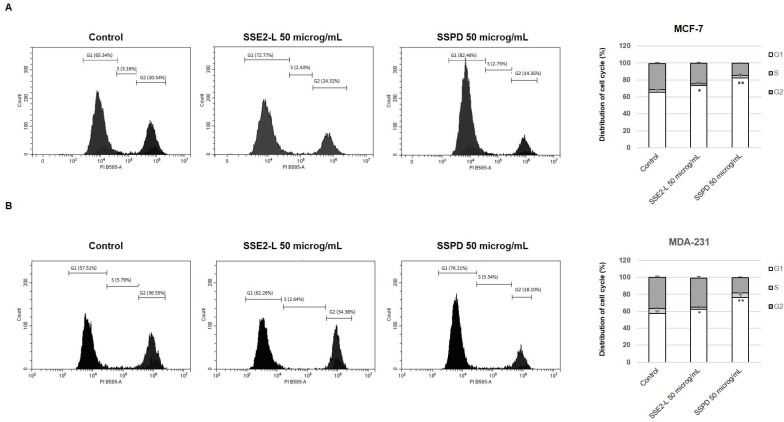
SSE2-L and SSPD effects on MCF-7 and MDA--MB 231 cell cycle population distribution. MCF-7 (**A**) and MDA—MB-231 (**B**) cells were left untreated (basal) or treated with SSE2-L or SPD for 24 h at 50 μg/mL. Cell cycle distribution was analyzed by flow cytometry following nuclear PI staining. The graphs on the right depict the percentage of MCF-7 and MDA-MB-231 cells within the different cell cycle phases. (* *p* < 0.05; ** *p* < 0.01 vs. basal).

After completing the anchorage-dependent MTT viability assay and the anchorage-independent soft agar assay, we next examined whether the compounds SSE2-L and SSPD could influence cell cycle progression. To this end, cells were stained with propidium iodide and analyzed by flow cytometry, allowing the distribution of the different cell cycle phases to be quantified (Figure 5). The analysis showed that exposure to both compounds resulted in an increased proportion of cells in the G1 phase, consistent with a block at this point of the cycle. While this effect was evident for both SSE2-L and SSPD, the accumulation in G1 was more pronounced in the samples treated with SSPD, suggesting a stronger impact of this compound on cell cycle arrest.

## 3. Discussion

The present work highlights the remarkable potential of pomegranate peel, an abundant agro-industrial by-product, as a renewable and sustainable source of high-value polyphenols with significant antioxidant and antiproliferative properties. With an environmentally friendly combined maceration–ultrasonication extraction approach, we successfully recovered an ethanolic extract enriched in hydrolysable tannins, notably punicalagin and ellagic acid derivatives, which displayed superior extraction yield, radical scavenging efficiency, and phenolic content compared to the acetonic counterpart. To address the inherent limitations of free pomegranate polyphenols, namely their chemical instability, low aqueous solubility, and limited bioavailability, a dextran-based polymeric conjugate (SSPD) via a mild aqueous-phase redox-initiated grafting reaction was developed. This strategy preserved the structural integrity of the bioactive compounds, endowed the conjugate with excellent antioxidant activity in both aqueous and organic environments, and provided a platform potentially capable of sustained release and enhanced delivery to biological targets. Dextran was chosen as the polymer backbone in this study owing to its excellent biocompatibility, biodegradability, non-immunogenicity and high water solubility [43]. Moreover, dextran has shown significant promise as an antitumour drug carrier: its abundant hydroxyl or oxidized functional groups allow chemical conjugation or encapsulation of cytotoxic agents, enabling enhanced tumor accumulation, prolonged circulation half-life, reduced systemic toxicity, and improved therapeutic efficacy relative to free drugs [44]. Comprehensive in vitro safety evaluations, including cytotoxicity (NRU assay), pro-sensitization (h-CLAT), and hemolysis testing, confirmed the biocompatibility of both the free extract (SSE2-L) and the dextran conjugate, with no evidence of adverse effects on fibroblasts, erythrocyte membranes, or monocyte immune activation. Importantly, both formulations exerted a marked, concentration- and time-dependent suppression of proliferation across two molecularly distinct human breast cancer cell lines, MCF-7 (ER^+^) and MDA-MB-231 (triple-negative). Proliferative capacity was first assessed using the anchorage-dependent MTT assay. Both SSE2-L and SSPD induced a clear, concentration- and time-dependent reduction in cell metabolic activity and viability, with SSPD consistently exerting the stronger inhibitory effect. The suppressive activity was evident at the earliest measurement points and became progressively more pronounced with increasing compound concentration and exposure duration, supporting the hypothesis that polymer conjugation enhances cellular uptake and/or local bioactive stability. The anti-proliferative capacity was further corroborated under anchorage-independent conditions by means of a soft agar colony formation assay. At 50 μg/mL, both compounds significantly reduced clonogenic potential in all tested cell lines, yet the inhibition was consistently greater in cells exposed to SSPD. The superior ability of SSPD to impair long-term clonal expansion in a three-dimensional environment underscores its enhanced efficacy in targeting hallmarks of tumorigenicity. To gain insight into the underlying mechanisms, cell cycle distribution was analyzed by flow cytometry following propidium iodide staining. Both SSE2-L and SSPD induced a measurable accumulation of cells in the G1 phase, consistent with a blockade at this checkpoint. Notably, the magnitude of G1 arrest was more pronounced in cells treated with SSPD, indicating a stronger capacity of the polymeric conjugate to interfere with regulatory pathways governing cell cycle progression. Taken together, these findings provide robust evidence that functionalization of pomegranate peel polyphenols into a dextran-based biopolymer matrix markedly enhances their anti-proliferative efficacy against breast cancer cells, without compromising safety or biocompatibility. The superior performance of SSPD relative to SSE2-L is likely attributable to improved physicochemical stability, higher effective concentrations at the cellular interface, and possibly prolonged exposure conferred by the polymeric carrier. Beyond oncology, the demonstrated antioxidant potency, favorable safety profile, and sustainable sourcing position these conjugates as promising candidates for broader biomedical and functional food applications. Future studies should aim to elucidate the molecular mechanisms involved, optimize conjugation parameters to maximize therapeutic benefit, and validate in vivo performance in relevant preclinical cancer models.

## 4. Materials and Methods

### 4.1. Chemicals

Folin-Ciocâlteu reagent, sodium nitrite (NaNO_2_), sodium molybdate (Na_2_MoO_4_), lipase, ferric chloride (FeCl_3_), dextran, sodium alginate and ammonium persulfate ((NH_4_)_2_S_2_O_8_) were purchased from Sigma Aldrich (St. Louis, MO, USA); acetone, methanol, dichloromethane, acetic acid, L-ascorbic acid and 30% hydrogen peroxide (H_2_O_2_) were purchased from Honeywell (Morristown, NJ, USA); sodium carbonate (Na_2_CO_3_) and sodium hydroxide (NaOH) were purchased from Carlo Erba (Milan, Lombardy, Italy); Aluminum chloride (AlCl_3_) and ABTS, IUPAC name 2,2′-azino bis(3-ethylbenzothiazoline-6-sulfonic acid), were purchased from Alfa Aesar (Haverhill, MA, USA); absolute ethanol was purchased from VWR Chemicals (Radnor, PA, USA); DPPH, IUPAC name 2,2′-diphenyl 1-picrylhydrazyl, was purchased from Tokyo Chemical Industry (Tokyo, Japan); 37% hydrochloric acid (HCl) was purchased from PanReac AppliChem (Barcelona, Spain).

### 4.2. Extractive Method

The pomegranate peels were collected and cleaned by quick immersion in a beaker containing warm (65–70 °C) distilled water and left to dry at room temperature, then cut into small pieces, and lyophilized (SSL). The extraction procedure was performed by a literature procedure with some modifications [25]. In detail, 1g of freeze-dried peels was mixed with 25 mL of extraction solvent (acetone and ethanol) and firstly macerated for 18 h a room temperature. After this, the same mixtures were sonicated for 40 min at 45 °C at 200 W, 40 KHz. The suspensions were filtered off with a Wathman filter and successively using a 0.22 μm membrane filter, to remove the solid particles. Both of the total yellow light extracts were evaporated and resulted in two light pink powders, namely SSA1-L 73.2 mg (when acetone was used as solvent) and SSE2-L 297.1 mg (when ethanol was used as solvent), respectively. The weighing operations were carried out using a Crystal 100 analytical balance by Gibertini (Milan, Lombardy, Italy). The sonication was carried out using an AU-32 ARGOLAB ultrasonic bath (Exacta Labcenter SpA, San Prospero (MO) Italia). The evaporation of the organic solvents, at the end of the extraction processes, was realized using a Laborota 4000 Efficient rotary evaporator by Heidolph (Schwabach, Germany). The centrifugation was conducted using an ALC Multispeed Centrifuge PK 121 centrifuge by Thermo Electron Corporation (Waltham, MA, USA).

### 4.3. Hydrolysable Tannins Evaluation

TLC using as eluent a mixture of methanol/dichloromethane/acetic acid 10:5:1, SSA1-L and SSE2-L were chromatographed and sprayed with a solution of 5% FeCl_3_. The blue coloration of the spots confirmed the presence of hydrolysable tannins in both extracts. The chromatographic column was performed using silica gel (0.040–0.063 mm) as the stationary phase, purchased from Merck (Darmstadt, Germany); for thin layer chromatography (TLC), ALUGRAM SIL G/UV254 plates purchased from Machery-Nagel (Düren, Germany) and TLC Silica gel 60 F254 glass-supported plates purchased from Merck (Darmstadt, Germany) were used. The reading of the thin-layer chromatography plates was carried out using a Spectroline Model CM-10 UV lamp by Spectronics Corporation (Melville, NY, USA).

### 4.4. ESI-MS/MS Analysis

The characterization of SSE2L and SSPD matrices was qualitatively performed by using an API 4000 Q-Trap mass spectrometer electrospray (MSD Sciex Applied Biosystem, Foster City, CA, USA) coupled to an HPLC 1200 system (Agilent Technologies, Santa Clara, CA, USA) in electrospray tandem mass spectrometry (ESI-MS/MS) in negative ion mode. Electrospray ionization tandem mass spectrometry (ESI-MS/MS) in negative ion mode was employed for the qualitative characterization of SSE2L and SSPD matrices. The optimized instrumental parameters used in direct infusion analysis (FIA) were the following: entrance potential (EP), −14 eV; declustering potential (DP), −80 eV; collision energy (CE) and collision exit potential (CXP), −27 and 11 eV, respectively. The separation of the analytes was carried out using a C18 Eclipse XDB-C8-A column (particle size of 5 μm, length of 150 mm and internal diameter of 4.6 mm) (Agilent Technologies).

### 4.5. Evaluation of the Phenolic Profile and Antioxidant Activity of Extracts

To assess the presence of polyphenols and evaluate their associated antioxidant activity, various colorimetric assays were performed on each extract obtained. The same procedures were performed on the dextran (DX) conjugate. The assays were conducted following specific protocols described in scientific literature.

#### 4.5.1. Determination of Total Polyphenol Content

The total polyphenol content (TPC) in both the extracts and the dextran (DX) conjugate was measured using the Folin–Ciocalteu method [45]. A precise amount of sample was weighed and dissolved in distilled water to prepare an aqueous solution at an appropriate concentration. From this stock solution, 6 mL was taken and mixed with 1 mL of Folin–Ciocalteu reagent. The mixture was left to stand in the dark for 3 min, after which 3 mL of sodium carbonate (Na_2_CO_3_) solution was added. The sample was then incubated in the dark for 2 h before its absorbance was recorded using a UV-Vis spectrophotometer at 760 nm. The absorbance readings were compared against a calibration curve created with gallic acid (GA) standards ranging from 8.0 to 40.0 μM to determine the TPC of each extract. Results were expressed as milligrams of gallic acid equivalents per gram of sample (mg GAE/g). For the analysis of the in vitro colorimetric assays, a UV-Vis Evolution 201 spectrophotometer by Thermo Fisher Scientific (Waltham, MA, USA) was used.

#### 4.5.2. Determination of Flavonoid Content in Extracts

The flavonoid content was determined following the procedure outlined by Medana et al. (2024) [21]. A measured amount of the sample was dissolved in distilled water to prepare an aqueous solution at an appropriate concentration. From this solution, 0.5 mL was taken and mixed with 2 mL of distilled water and 0.15 mL of a 15% aqueous sodium nitrite (NaNO2) solution. The mixture was kept in the dark for 6 min. Then, 0.15 mL of a 10% (*w*/*v*) aqueous aluminum chloride (AlCl3) solution was added, and the tube was again left in the dark for another 6 min. Following this, 2 mL of a 4% (*w*/*v*) sodium hydroxide (NaOH) solution and additional distilled water were added to reach a final volume of 5 mL. The sample was incubated in the dark for a further 15 min before measuring absorbance at 510 nm using a UV-Vis spectrophotometer. The absorbance values and sample concentrations were analyzed against a (+)-catechin (CT) calibration curve to quantify flavonoid content in each extract. Results were expressed as milligrams of catechin equivalents per gram of sample (mg CTE/g).

#### 4.5.3. DPPH Assay

The radical scavenging activity against 2,2′-diphenyl-1-picrylhydrazyl (DPPH) was evaluated according to the procedure described by Fedeli et al. (2024) [46]. The DPPH• reagent was prepared by dissolving 7.8 mg of the radical in 100 mL of absolute ethanol and stored in the dark until use. A known amount of sample was dissolved in alcohol to prepare solutions at various concentrations. For the assay, 5.0 mL of each diluted sample solution was mixed with 5.0 mL of the DPPH• reagent. The mixtures were then incubated in the dark for 20 min, after which their absorbance was recorded at 517 nm using a UV-Vis spectrophotometer. The percentage inhibition of the DPPH• radical was calculated using the formula:

% Inhibition = (*A*_0_ − *A*_1_):*A*_0_ × 100 (1)

where *A*_0_ is the absorbance of the control (without sample) and *A*_1_ is the absorbance of the sample. The antioxidant capacity of each extract was expressed as the IC_50_ value, representing the concentration required to inhibit 50% of the DPPH• radicals. Ascorbic acid was used as a positive control.

#### 4.5.4. ABTS Assay

The antioxidant activity against the 2,2′-azino-bis(3-ethylbenzothiazoline-6-sulphonic acid) (ABTS) radical was assessed following the procedure reported by Aiello et al. (2024) [47]. A stock solution was prepared by dissolving 38 mg of ABTS and 5.6 mg of ammonium persulfate in distilled water within a 10 mL volumetric flask. The ammonium persulfate served to oxidize ABTS, generating the ABTS^+^• cation radical. This solution was kept in the dark for 24 h before dilution; subsequently, 1.0 mL of the stock was diluted with 35 mL of distilled water. Samples were dissolved at appropriate concentrations, and, in a typical assay, 0.5 mL of sample solution at various concentrations was combined with 2.0 mL of the ABTS^+^• reagent. After incubation in the dark for 6 min, the absorbance was measured at 734 nm using a UV-Vis spectrophotometer. The absorbance data were then used to calculate the percentage inhibition of the ABTS radical according to Equation (1). Antioxidant activity for each sample was expressed as the IC_50_ value, indicating the concentration needed to inhibit 50% of the ABTS radicals. Ascorbic acid was used as a positive control.

### 4.6. Synthesis of Dextran-Based Polymeric Conjugate via Grafting Reaction

The DX conjugate was prepared through a grafting reaction based on the method reported by Aiello et al. (2023) [41], with some adjustments. To start, 0.500 g of DX was dissolved in 25.0 mL of distilled water under continuous magnetic stirring. Then, 12.5 mL of hydrogen peroxide (120 vol) and 0.250 g of ascorbic acid were added to the reaction mixture. After stirring for 2 h, 0.472 g of SSE2-L extract, previously dissolved in 12.5 mL of distilled water, was introduced, and the mixture was stirred overnight. The resulting reaction solution was purified by dialysis using a membrane with a molecular weight cut-off (MWCO) of 3.5 kDa against distilled water for 72 h. Finally, the purified solution was freeze-dried, yielding a light brown, fluffy solid designated as SSPD. A control polymer sample, named BDX, was also prepared following the same protocol but without the addition of SSE2-L extract. The freeze-drying operations were conducted with a Micro Modulyo freeze-dryer by Edwards Lifescience (Irvine, CA, USA). A dialysis membrane with a diameter of 11.5 mm and a MWCO of 3.5 kDa purchased from Spectrum Laboratories (Piscataway, NJ, USA) was used.

### 4.7. Cell Cultures

The MDA-231, MCF-7, BALB/3T3, and THP-1 cell lines used in the experimental work were maintained in culture as follows:

MCF-7 and MDA-MB-231 cell lines were obtained from the American Type Culture Collection (ATCC, Manassas, VA, USA) and cultured in DMEM/F12 medium (Sigma-Aldrich, St. Louis, MO, USA) supplemented with 10% fetal bovine serum (FBS; Sigma-Aldrich), 2 mM L-glutamine (Sigma-Aldrich), and 1% penicillin/streptomycin (Sigma-Aldrich).

BALB/3T3 cells, murine fibroblasts, were cultured in DMEM-High Glucose 1X medium supplemented with 10% bovine calf serum (BCS), 1% penicillin/streptomycin, and 1% sodium pyruvate.

THP-1 cells, a monocytic cell line derived from the peripheral blood of a patient with acute monocytic leukemia, were cultured in RPMI-1640 medium supplemented with 10% fetal bovine serum (FBS), 1% penicillin/streptomycin, and 0.05 mM β-mercaptoethanol.

All cell lines were maintained at 37 °C in a humidified atmosphere containing 5% CO_2_.

### 4.8. Safety Testing

#### 4.8.1. Neutral Red Uptake Assay

The Neutral Red Uptake (NRU) assay was employed to assess the in vitro cytotoxic potential of the test compound as an indicator of biocompatibility. This colorimetric assay, assessed according to the guidelines ISO 10993-5:2009 [39] and ISO 10993-12:2021 [40], exploits the capacity of viable cells to incorporate the supravital dye neutral red into lysosomes via active transport, a function that is impaired in non-viable or damaged cells.

Murine fibroblast BALB/3T3 clone A31 cells were seeded in 96-well flat-bottom microplates at a density of 1 × 10^4^ cells/well in complete culture medium and incubated for 24 h at 37 °C in a humidified atmosphere of 5% CO_2_. Subsequently, the culture medium was replaced with fresh medium containing SSE2-L and SSPD at graded concentrations, alongside sodium dodecyl sulfate (SDS, positive control) and untreated cells (negative control). Plates were returned to the incubator under identical conditions for an additional 24 h.

Following exposure, the treatment medium was aspirated and replaced with neutral red working solution. Cells were incubated for 3 h at 37 °C, 5% CO_2_. Thereafter, the dye-containing medium was removed, and cells were gently rinsed with phosphate-buffered saline (PBS) to eliminate unincorporated dye. A solubilization solution (ethanol/acetic acid/deionized water) was then added to each well to extract the incorporated dye, and plates were subjected to gentle orbital shaking for 10 min. Optical density (OD) was determined spectrophotometrically at 540 nm through a Synergy H1 microplate reader (Hybrid Reader, BioTek Instruments, Agilent, CA, USA.

Each experimental condition was tested in triplicate in three independent experiments. Cell viability (%) was calculated relative to the negative control, which was set as 100%. According to ISO 10993-5:2009 guidelines, samples exhibiting cell viability ≥ 70% were classified as non-cytotoxic, whereas those with viability < 70% were considered cytotoxic.

#### 4.8.2. Human Cell Line Activation Test (h-CLAT)

The human cell line activation test (h-CLAT) was conducted following the principles described in OECD Test Guideline 442E, which assesses whether a chemical has the potential to activate dendritic cells, an essential step in the skin sensitization pathway. The approach relies on detecting variations in the expression of the co-stimulatory markers CD54 and CD86 on THP-1 human monocytic cells by flow cytometry. Nickel sulfate (NiSO_4_, 100 μg/mL) served as the positive control, while untreated complete culture medium was used as the negative control.

THP-1 cells were cultured in RPMI-1640 supplemented with 10% heat-inactivated fetal bovine serum (FBS), 1% penicillin-streptomycin, and 0.05 mM β-mercaptoethanol, and maintained at 37 °C in a humidified 5% CO_2_ incubator. Cells were seeded into 96-well plates at 1.5 × 10^5^ cells/well and pre-incubated for 24 h before exposure. Cytotoxicity was preliminarily evaluated by propidium iodide (PI) exclusion using flow cytometry, and the concentration reducing viability by 25% (CV_75_) was determined. The highest non-toxic level tested corresponded to 1.2 × CV75. A 100× stock solution of the test item was prepared in phosphate-buffered saline (PBS) and further serially diluted (1:1.2 ratio) to produce three additional concentrations spanning from 0.335 × CV75 to 1.2 × CV75. These were diluted 1:50 in complete medium immediately before treatment and then applied to the cells at a further 1:2 dilution.

Following 24 h exposure, cells were harvested by centrifugation, washed in fluorescence-activated cell sorting (FACS) buffer, and split into three aliquots for staining. Non-specific binding was minimized by pre-incubating the cells with a blocking solution containing 0.01% γ-globulins in FACS buffer for 15 min at 4 °C. Cells were then labeled with fluorescein-conjugated antibodies against CD54, CD86, or the IgG1 isotype control for 30 min at 4 °C. After washing, cells were resuspended in FACS buffer containing PI to allow concurrent viability assessment.

Flow cytometry was performed to quantify CD54 and CD86 surface expression. Data analysis excluded debris and PI-positive events, and median fluorescence intensity (MFI) values were normalized against the isotype control to obtain relative fluorescence intensity (RFI). In line with OECD TG 442E interpretation criteria, a sample was considered positive when CD86 RFI was ≥150% and/or CD54 RFI was ≥200%, provided cell viability exceeded 50%.

#### 4.8.3. Hemolysis Assay

Fresh peripheral venous blood was collected from healthy donors into 4 mL sodium citrate-treated vacutainer tubes to prevent coagulation. Erythrocytes were isolated by centrifugation at 2000 rpm for 10 min at 4 °C, according to a previously established protocol [48]. The red blood cell (RBC) pellet was washed three times with ice-cold phosphate-buffered saline (PBS, pH 7.4) to remove plasma and buffy coat components and subsequently diluted 10-fold in PBS to obtain a 10% (*v*/*v*) erythrocyte suspension and aliquoted at 1 mL per tube.

Aliquots of the RBC suspension were incubated with SSE2-L (25 or 100 μg/mL) or SSPD (25 or 100 μg/mL) for 24 h at 37 °C under gentle agitation. Following incubation, samples were centrifuged at 2000 rpm for 10 min at 4 °C, and hemoglobin release in the supernatant was quantified spectrophotometrically at 540 nm using a Synergy H1 microplate reader (BioTek). Measurements were performed at three time points: 1 h, 6 h, and 24 h post-treatment.

A 0.1% (*v*/*v*) Triton X-100 solution was employed as a positive control to induce complete erythrocyte lysis (100% hemolysis), whereas PBS served as a negative control. All experiments involving human blood were conducted in accordance with the Declaration of Helsinki, with informed donor consent, and were approved by the Ethics Committee of the University of Calabria [49].

### 4.9. Efficacy Testing

#### 4.9.1. MTT Assay

Cell viability was evaluated using the MTT [3-(4,5-dimethylthiazol-2-yl)-2,5-diphenyltetrazolium bromide] colorimetric assay, which quantifies mitochondrial metabolic activity as an indirect measure of viable, adherent cell number. The method relies on the enzymatic reduction of the yellow tetrazolium salt MTT to insoluble purple formazan crystals by mitochondrial dehydrogenases in metabolically active cells. This conversion occurs exclusively in viable cells, reflecting intact mitochondrial function.

MCF-7 and MDA-231 cells were cultured in 48-well plates and exposed to the test formulations, consisting of SSE2-L and SSPD at increasing concentrations for 24 or 48 h. Following the treatment period, 200 μL of MTT solution was added to each well, and the plates were incubated for 2 h at 37 °C in a humidified environment.

#### 4.9.2. Flow Cytometric Evaluation of DNA Content

MCF-7 and MDA-231 cells were cultured in six-well plates and exposed to SSE-L and SSPD (50 μg/mL) for 24 h. Following treatment, cells were detached by trypsinization and resuspended in 0.5 mL of DNA staining solution containing propidium iodide (0.1 mg/mL), sodium citrate (0.1%), Triton X-100 (0.1%), and RNase (0.02 mg/mL; Sigma). DNA content was quantified using a CytoFLEX flow cytometer (Beckman Coulter SRL, Milan, Italy). For each sample, 10,000 nuclei were recorded, and the distribution of cells across the G1, S, and G2/M cell cycle phases was determined using CytExpert software (version 2.6, Beckman).

#### 4.9.3. Soft-Agar Colony Formation Assay

Cells were plated in 12-well dishes and cultured either with or without SSE-L or SSPD at the specified concentrations for 14 days. At the end of the incubation period, colonies were visualized by staining with 0.05% Coomassie Blue prepared in a methanol/water/acetic acid solution (45:45:10, *v*/*v*; Sigma). Colony numbers were subsequently counted and expressed relative to untreated control samples.

### 4.10. Statistical Analysis

All in vitro statistical analyses were conducted using GraphPad Prism software, version 8 (GraphPad Software, San Diego, CA, USA). Data are presented as mean ± standard deviation (SD) from at least three independent experiments. Differences between the two experimental groups were evaluated using an unpaired two-tailed Student’s *t*-test. A *p*-value less than 0.05 was considered statistically significant.

## Figures and Tables

**Table 1:** Phenolic profile and antioxidant activity of the pomegranate extracts.

Extract	TPC (mg GAE/g)	FC (mg CTE/g)	Scavenger Activity IC_50_ (mg mL^−1^)	
			DPPH	ABTS
**SSA1**	19.53 ± 0.84 ^a^	65.21 ± 2.87 ^b^	0.1982 ± 0.0081 ^d^	0.0482 ± 0.0018 ^c^
**SSE2**	19.43 ± 0.89 ^a^	31.32 ± 1.24 ^a^	0.1821 ± 0.0084 ^c^	0.0510 ± 0.0020 ^c^
**SSA1-L**	466.12 ± 16.24 ^c^	365.23 ± 15.41 ^d^	0.0186 ± 0.0008 ^b^	0.0093 ± 0.0004 ^a^
**SSE2-L**	318.08 ± 12.85 ^b^	177.33 ± 7.46 ^c^	0.0159 ± 0.0005 ^a^	0.0191 ± 0.0009 ^b^
**Positive control**				
***Ascorbic acid***			0.0017 ± 0.0004	0.0005 ± 0.0008

GAE = Gallic Acid Equivalent; CTE = (+)-Catechin Equivalent; TPC = Total Phenol Content; FC = Flavonoid Content; DPPH = 2,2′-diphenyl-1-picrylhydrazyl radical; ABTS = 2,2′-azino-bis(3-ethylbenzothiazoline-6-sulphonic acid) radical. Different letters are significantly different at *p* < 0.05.

**Table 2:** Expression of co-stimulatory molecules in THP-1 monocytes after 24 h exposure to test samples.

Sample	CD86 *	CD54 *
SSE2-L 50 μg/mL	57	71
SSPD 50 μg/mL	52	69
Control	35	59
Positive Control (NISO_4_)	191	236

* Cut-off values: CD86 > 150; CD54 > 200.

## Data Availability

The original contributions presented in this study are included in the article/Supplementary Material. Further inquiries can be directed to the corresponding author.

## Supplementary Materials

The following supporting information can be downloaded at: https://www.mdpi.com/article/10.3390/ijms262110618/s1.
