# Supplementary material for: Antiproliferative Evaluation of Dextran Polymer-Based Pomegranate Ethanolic Extract
Source: Int J Mol Sci. 2025 Oct 31;26(21):10618. doi: 10.3390/ijms262110618 (PMC12608317; doi:10.3390/ijms262110618)

SUPPLEMENTARY MATERIAL SECTION

Antiproliferative Evaluation of Dextran Polymer-Based Pomegranate Ethanolic Extract

Umile Gianfranco Spizzirri [1], Marisa Francesca Motta [2], Sonia Ferraro [2], Silvia Strigaro [2], Cinzia Benincasa [3], Rosa Nicoletti [3], Francesco Astuto [1], Ubaldo Comite [4], Rocco Malivindi [2,5,*] and Francesca Aiello [2]

[1] Ionian Department of Law, Economics and Environment, University of Bari Aldo Moro, 74123 Taranto, Italy; umile.spizzirri@uniba.it (U.G.S.); astutof22@gmail.com (F.A.)
[2] Department of Pharmacy, Health and Nutritional Sciences, University of Calabria, Edificio Polifunzionale, 87036 Rende, Italy; marisafrancesca.motta@unical.it (M.F.M.); ferraro24sonia@gmail.com (S.F.); silvia.strigaro@libero.it (S.S.); francesca.aiello@unical.it (F.A.)
[3] Council for Agricultural Research and Economics (CREA), Research Centre for Olive, Fruit and Citrus Crops, Via Settimio Severo 83, 87036 Rende, Italy; cinzia.benincasa@crea.gov.it (C.B.); rosa.nicoletti@crea.gov.it (R.N.)
[4] Department of Business Sciences, University Giustino Fortunato, 82100 Benevento, Italy; u.comite@unifortunato.eu
[5] Clinical Laboratory Unit, AO SS Annunziata, 87100 Cosenza, Italy
* Correspondence: rocco.malivindi@unical.it

Correspondence to

Rocco Malivindi: rocco.malivindi@unical.it

Figure S1. ESI-MS/MS spectrum of SSE2L

Figure S2. ESI-MS/MS spectrum of SSPD

Figure S3. ESI-MS/MS spectrum of BDX

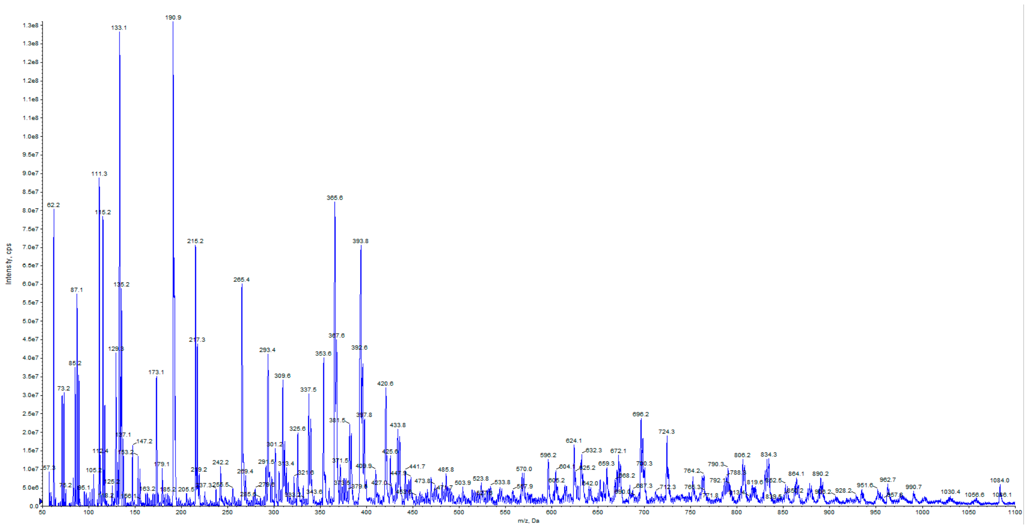

Figure S1. Full ion scan mass spectrum of pomegranate extract (SSE2L).

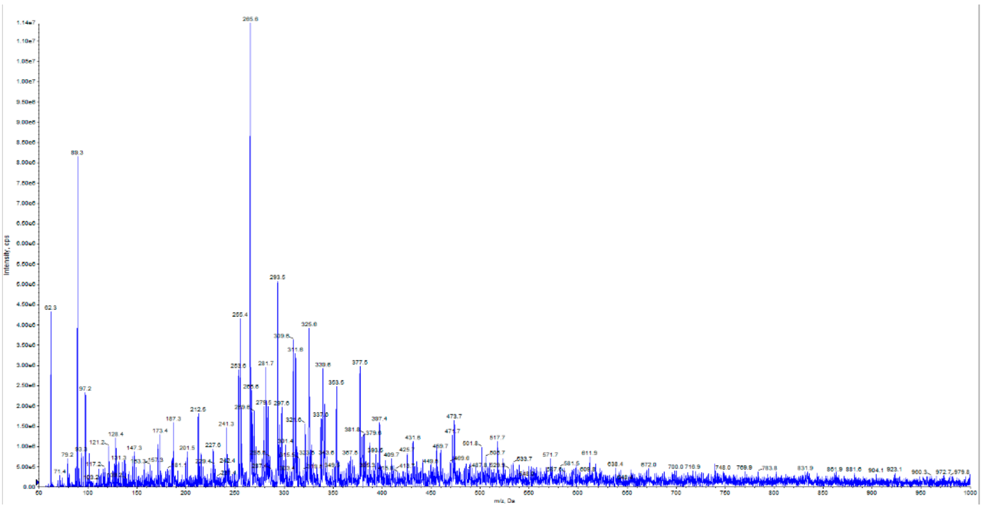

Figure S2. Full ion scan mass spectrum of the functionalized polymer (SSPD).

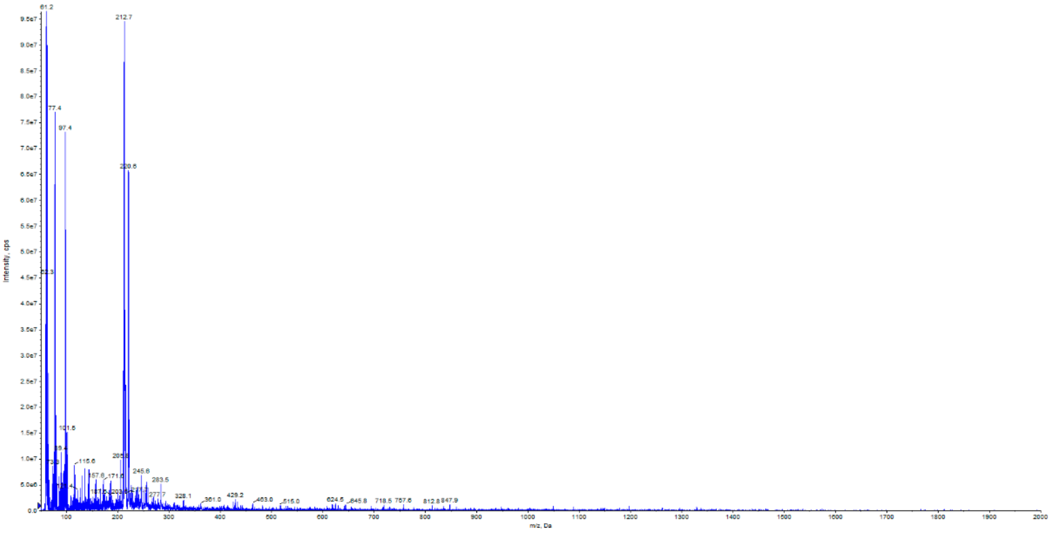

Figure S3. Full ion scan mass spectrum of dextran polymer synthesized without extract (BDX).

Table S1. Deprotonated molecular ions and main fragments.

[M-H]$^-$ (m/z)	Main fragments (m/z)	Compound
265.2	97.0	Gallic acid derivative
293.1	97.1; 79.0	Gallic acid derivative
309.3	97.3; 122.8; 80.0	Gallic acid derivative
325.3	183.2; 196.8; 119.1	Coumaric acid derivative
339.4	193.2; 163.0; 196.8	Coumaric acid derivative
377.0	341.0; 178.7; 119.3	Coumaric acid derivative
397.3	97.0; 351.7	Gallic acid derivative

Table S2. Results interpretation.

Score	*Reactivity*	*Condition of All Cultures*
0	None	*No alterations*
1	Slight	*Presence of some altered cells under the sample*
2	Mild	*Alteration present in a limited area under the sample*
3	Moderate	*Alteration present in extending area under the sample up to 1.0 cm*
4	Severe	*Area extending more than 1.0 cm outside the sample*

Table S3. Assessment of biological reactivity.

**Sample**	**Biological Reactivity**
Control	0
SSE2-L 12,5 µg/ml	1
SSE2-L 25 µg/ml	0
SSE2-L 50 µg/ml	0
SSE2-L 75 µg/ml	1
SSE2-L 100 µg/ml	1
SSPD 12,5 µg/ml	0
SSPD 25 µg/ml	0
SSPD 50 µg/ml	0
SSPD 75 µg/ml	1
SSPD 100 µg/ml	1
Control + (SDS 10%)	4

Figure S4: Histograms shown the fluorescence intensity percentage of CD54 and CD86 evaluated by flow cytometry for the hCLAT test.

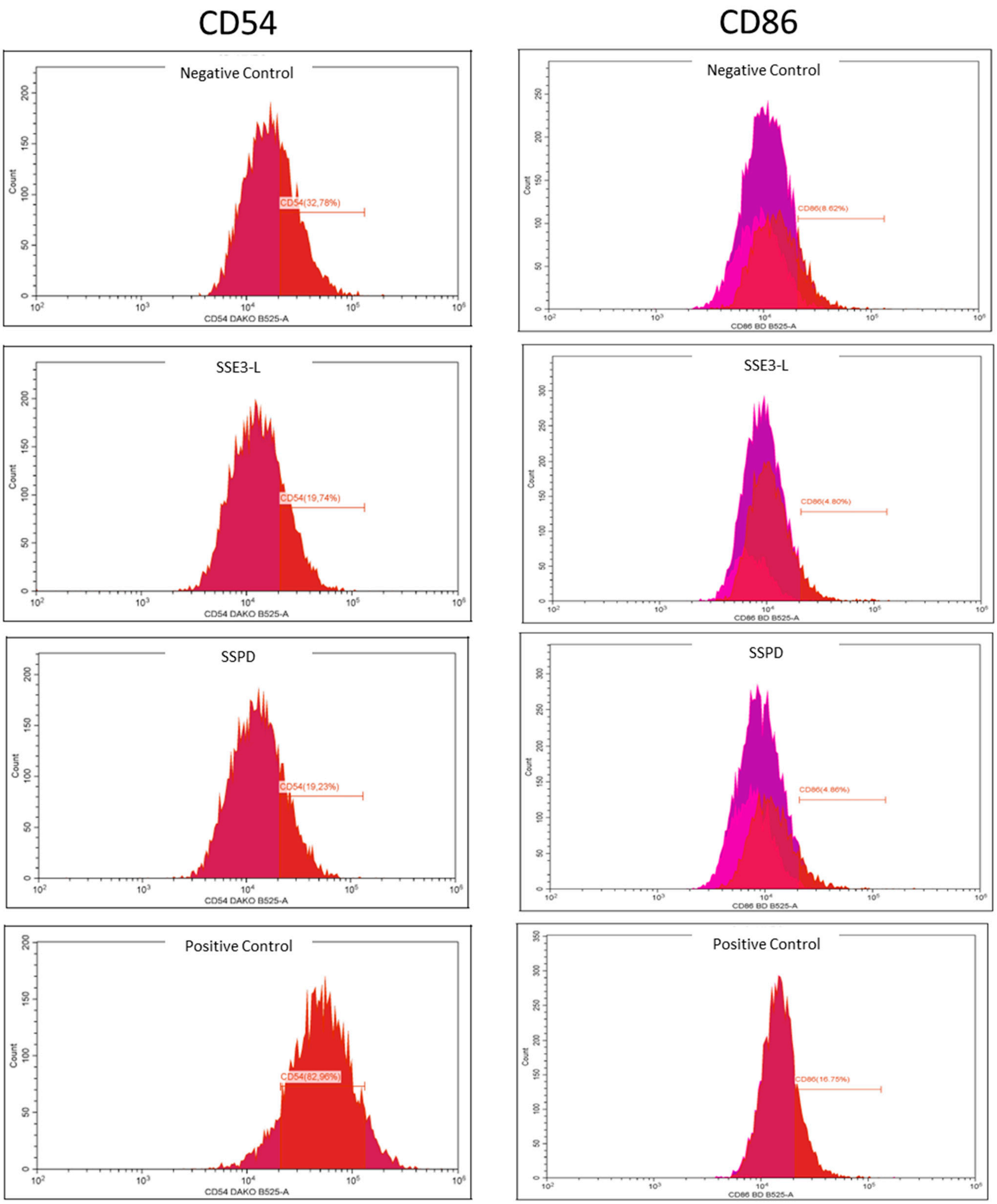

Supplement: Supplementary file 1 [file ijms-26-10618-s001.zip › ijms-3868287-supplementary.pdf]
